# Neurovascular Coupling During Visual Stimulation in Multiple Sclerosis: A MEG-fMRI Study

**DOI:** 10.1016/j.neuroscience.2018.03.018

**Published:** 2019-04-01

**Authors:** Rachael Stickland, Marek Allen, Lorenzo Magazzini, Krish D. Singh, Richard G. Wise, Valentina Tomassini

**Affiliations:** a Cardiff University Brain Research Imaging Centre (CUBRIC), Cardiff University School of Psychology, Maindy Road, Cardiff CF24 4HQ, UK; b Institute of Psychological Medicine and Clinical Neurosciences, Cardiff University School of Medicine, University Hospital Wales, Heath Park, CF14 4XN, UK

**Keywords:** ASL, arterial spin labeling, BOLD, blood oxygen level dependent, CBF, cerebral blood flow, fMRI, functional Magnetic Resonance Imaging, GM, gray matter, GG, Greenhouse–Geisser, MAR, MAgnification Requirement, MEG, magnetoencephalography, MS, Multiple Sclerosis, PASAT, Paced Auditory Serial Addition Test, ROI, region of interest, SEM, Standard Error of the Mean, T25-FW, Time 25 Foot Walk, TI, inversion time, VEF, visual-evoked field, WM, white matter, 9-HPT, Nine-Hole Peg Test, Multiple Sclerosis, neurovascular coupling, functional MRI, cerebral blood flow, magnetoencephalography, visual function

## Abstract

The process of neurovascular coupling ensures that increases in neuronal activity are fed by increases in cerebral blood flow. Evidence suggests that neurovascular coupling may be impaired in Multiple Sclerosis (MS) due to a combination of brain hypoperfusion, altered cerebrovascular reactivity and oxygen metabolism, and altered levels of vasoactive compounds.

Here, we tested the hypothesis that neurovascular coupling is impaired in MS. We characterized neurovascular coupling as the relationship between changes in neuronal oscillatory power within the gamma frequency band (30–80 Hz), as measured by magnetoencephalography (MEG), and associated hemodynamic changes (blood oxygenation level dependent, BOLD, and cerebral blood flow, CBF) as measured by functional MRI. We characterized these responses in the visual cortex in 13 MS patients and in 10 matched healthy controls using a reversing checkerboard stimulus at five visual contrasts.

There were no significant group differences in visual acuity, P100 latencies, occipital gray matter (GM) volumes and baseline CBF. However, in the MS patients we found a significant reduction in peak gamma power, BOLD and CBF responses. There were no significant differences in neurovascular coupling between groups, in the visual cortex.

Our results suggest that neuronal and vascular responses are altered in MS. Gamma power reduction could be an indicator of GM dysfunction, possibly mediated by GABAergic changes. Altered hemodynamic responses confirm previous reports of a vascular dysfunction in MS. Despite altered neuronal and vascular responses, neurovascular coupling appears to be preserved in MS, at least within the range of damage and disability studied here.

## Highlights

• A reduced electrophysiological response to a visual stimulus in MS, characterized by reduced gamma power (30–80 Hz), with MEG.
• A reduced hemodynamic response to a visual stimulus in MS, characterized by reduced BOLD and CBF responses, with fMRI.
• The coupling between gamma power and BOLD/CBF was not significantly impaired in the MS group.

## Introduction

In the healthy brain, neuronal activity and cerebral blood flow (CBF) have a close spatial and temporal relationship: increases in neuronal activity are associated with local increases in CBF via changes in blood vessel tone, a process known as neurovascular coupling. Neurovascular coupling is mediated by the interaction of neuronal, glial and vascular cells (Attwell and Iadecola, 2002, Girouard and Iadecola, 2006, Attwell et al., 2010, Petzold and Murthy, 2011) and is thought to be impaired in many neurovascular and neurodegenerative conditions (Iadecola, 2004, Girouard and Iadecola, 2006, Cantin et al., 2011). As neurovascular coupling is a key physiological mechanism in the heathy brain, its alteration in disease is thought to contribute to tissue dysfunction and damage.

In Multiple Sclerosis, hypoperfusion is seen in both GM and normal appearing WM (Swank et al., 1982, Brooks et al., 1984, Lycke et al., 1993, Sun et al., 1998, Law et al., 2004, Adhya et al., 2006, D’haeseleer et al., 2013), as well as reports of impaired vascular reactivity (Marshall et al., 2014, Marshall et al., 2016) and reduced oxygen metabolism (Ge et al., 2012). Vasoactive agents such as nitric oxide and endothelin-1 that have profound, and often contrasting, effects on the vasculature are significantly raised within MS lesions (Smith and Lassmann, 2002, D’haeseleer et al., 2013). Glial cells have a key role in responding to damage in the MS brain, as well as playing a crucial role in neurovascular coupling (Metea and Newman, 2006). The combination of these factors may lead to an alteration of the hemodynamic response to neuronal activity in MS, the hypothesis tested in this study.

Here, we investigated neurovascular coupling in MS using two complementary non-invasive imaging modalities: magnetoencephalography (MEG) and functional Magnetic Resonance Imaging (fMRI). fMRI signals are based on the local vascular response, a process known as functional hyperaemia. MEG directly measures magnetic fields generated by the electrical currents produced by synchronous activity of thousands of neurons, the local field potential (Hansen et al., 2010). Both MEG and fMRI signals are thought to largely reflect postsynaptic (dendritic) rather than axonal activity (Logothetis, 2002, Zhu et al., 2009, Hall et al., 2014). Commonly, when cortical networks are activated there is an increase in the signal power of faster oscillations, i.e. in the gamma band (Demanuele et al., 2007, Jia and Kohn, 2011). Most findings show a positive correlation between changes in gamma band activity (typically >30 Hz) and the hemodynamic response (Mukamel et al., 2005, Niessing et al., 2005, Zumer et al., 2010), as well as good spatial coherence between these signals (e.g., Singh et al., 2002, Muthukumaraswamy and Singh, 2008).

By displaying the same reversing checkerboard stimulus at five levels of contrast, we probed neuronal and hemodynamic responses in the visual cortex, expecting these responses in the early visual areas to increase monotonically with increasing contrast (Goodyear and Menon, 1998, Hall et al., 2005, Henrie and Shapley, 2005, Muthukumaraswamy and Singh, 2009, Perry et al., 2015). We used the positive relationship between gamma power (30–80 Hz) and hemodynamic signals as our empirical measure of neurovascular coupling.

MEG studies investigating MS have mostly used resting-state paradigms, showing clear network disruption across theta, alpha and beta frequency bands (e.g., Cover et al., 2006; Schoonheim et al., 2015; Van der Meer et al., 2013, Tewarie et al., 2013, Tewarie et al., 2014, Tewarie et al., 2015). At the time of this research, no studies have reported on gamma oscillatory changes in MS. Given possible GM dysfunction and damage, we predicted a reduction in gamma power in the MS group. Based on the vascular impairments reported in MS, we predicted that the MS group would have a reduced hemodynamic response to stimulation, and that neurovascular coupling in the visual cortex would be altered.

We report no significant group differences in visual acuity scores, P100 latencies, occipital GM volumes and baseline CBF. However, in the MS patients we found a significant reduction in peak gamma power, the blood oxygen-level-dependent (BOLD) response and CBF response to visual stimulation. The patient group presented with more varied neurovascular coupling relationships than the controls, but there was no significant group difference in neurovascular coupling, in the early visual cortex.

## Experimental procedures

### Subjects

Patients with a diagnosis of MS (Polman et al., 2011) were recruited at the University Hospital Wales, Cardiff. Patients were treatment naïve, but eligible to start first-line disease-modifying treatment and had not experienced a relapse in the last 3 months. Age- and gender-matched healthy controls were recruited. Written consent was obtained according to the protocol approved by Research Ethics Committee, Wales, UK.

### Testing sessions

All participants had a behavioral session, a MEG and an MRI scan performed on the same day, except for one control who returned on a different day for the MRI scan.

#### Behavioral testing

Patients’ disability was assessed using the *Expanded Disability Status Scale (EDSS)* (Kurtzke, 1983). Tests from the MS Functional Composite (Cutter et al., 1999) were carried out on the patients and controls: *Nine-Hole Peg Test (9-HPT)* for upper limb motor function, the *Timed 25 Foot Walk (T25-FW)* for mobility and walking, and the *Paced Auditory Serial Addition Test (PASAT) 2 and 3 s* as a measure of sustained attention. Visual acuity was assessed, in each eye separately, with a SLOAN letter chart (Precision Vision) at 100%, 25%, 10%, 2.5%, 1.25% and 0.6% contrast, expressed as a decimal that represented viewing distance divided by the letter size (in M-units). All participants except five required corrective lenses for daily use and wore them throughout the testing sessions.

#### Visual paradigm during scanning

Identical stimulation parameters were used for fMRI and MEG. The visual stimulus consisted of a black and white checkerboard, polarity reversing every 250 ms. Checks were squares, with a spatial frequency of 1 cycle per degree. The checkerboard was displayed on a mean luminance background, with a small red fixation circle in the center. The rest conditions consisted only of this background and fixation. For both scanning modalities, the entire stimulus field was 16 × 16° of visual angle and the stimulus was projected on screens with a 1024 × 768 resolution and 60-Hz refresh rate. The checkerboard was displayed at 5 Michelson contrast levels: 6.25%, 12.5%, 25%, 50% and 100%. Stimuli were displayed in 30-s blocks and each contrast level was presented 4 times. The rest blocks were also 30 s long, but were presented 8 times. The block order was pseudorandomized across participants, but for each participant the same block order was used for both MEG and fMRI. The task lasted for 14 min and was repeated twice, once for each eye, with the untested eye covered with a cotton pad. We tested separate eyes because a common initial presentation of MS is optic neuritis, an acute, often unilateral, visual impairment characterized by a reduction in visual acuity and connectivity in visual pathways (Polman et al., 2011, Toosy et al., 2014). The experiments were programmed in MATLAB, using the Psychophysics Toolbox extensions (Kleiner et al., 2007).

#### MEG data acquisition

A 275-channel CTF axial gradiometer system was used to obtain whole-head MEG recordings, sampled at 1200 Hz (0- to 300-Hz band-pass). An additional 29 reference channels were recorded for noise cancelation, and 3 of the 275 channels were turned off due to excessive sensor noise. Fiduciary coils were placed at fixed distances from three anatomical landmarks (nasion, left, and right pre-auricular) and the positions of the coils were monitored continuously. For co-registration, these landmarks were later identified on the subject’s structural MRI and also verified with digital photographs. The MEG data were acquired continuously and epoched offline.

#### MRI data acquisition

MRI data were acquired on a 3T GE HDx MRI system using an eight-channel receiver head coil. A 3D T1-weighted structural scan was obtained for each participant: fast-spoiled gradient recalled echo (FSPGR): acquisition matrix = 256 × 256 × 172, 1 × 1 × 1 mm voxels, TE = 2.9 ms, TR = 7.8 ms.

During the visual task, a pulsed arterial spin labeling (ASL) scan sequence was acquired with a dual gradient-echo spiral k-space readout (TR/TE1/TE2 = 2200/3/29 ms, 64 × 64 × 12 slices, voxels 3.4 × 3.4 × 7 mm, 1-mm inter-slice gap, ascending order, 22-cm field of view in-plane, flip angle 90°), the first echo being used to estimate CBF changes and the second echo being used for BOLD time series analysis. The proximal inversion and control for off-resonance effects (PICORE) labeling scheme was used, with a label thickness of 20 cm (TI1 = 700 ms, TI2 = 1600 ms for most proximal slice) and 10-mm gap between labeling slab and bottom slice. An adiabatic hyperbolic secant inversion pulse was used with quantitative imaging of perfusion using a single subtraction (QUIPSS II), with a 10-cm saturation band thickness (Wong et al., 1998). 191 tag-control pairs resulted in 382 volumes being acquired over the 14-min task. While the participant was at rest, two single-echo multi inversion time, MTI, (post label delay) pulsed ASL scans (Chappell et al., 2010) were acquired in order to estimate baseline perfusion (scan 1, inversion times (TI): 400, 500, 600, 700 ms, scan 2, TIs: 1000, 1100, 1400, 1700 and 2000 ms). The same PICORE labeling sequence was used as explained above, with a QUIPSS II cut of at 700 ms for TIs > 700 ms. A variable repetition time was used in order to minimize scan time. 16 tag-control pairs for each TI were acquired.

Before both pulsed ASL scans, a calibration scan was acquired in order to obtain the equilibrium magnetization (*M*_0_) of cerebrospinal fluid for the purposes of perfusion quantification: a single volume with the same acquisition parameters but without the ASL preparation and with an effectively infinite TR (so magnetization fully relaxed). Additionally, a minimum contrast scan was acquired to correct for received image intensity variation with the same previous parameters, except TE = 11 ms, TR = 2 s, and 8 interleaves.

### Data analysis

#### Behavioral data analysis

The 9-HPT, T25-FW, PASAT-2, and PASAT-3 were all scored with the BRB-N manual (Brief Repeatable Battery of Neuropsychological Tests in Multiple Sclerosis). Responses from 9-HPT and the T25-FW were measured in seconds to complete, and the PASAT in number of correct trials. For visual acuity, the MAR value (**MA**gnification **R**equirement – the inverse of the visual acuity score) was calculated. Values were then expressed in log(MAR) units, which indicate “visual acuity loss”. A value of 0 indicates no loss so is equivalent to visual acuity at the reference standard (20/20), and an increment increase of 0.1 log(MAR) indicates one line of loss.

#### MEG data analysis

The analysis of MEG data was performed in MATLAB using the Fieldtrip toolbox (Oostenveld et al., 2011). First, data segments including large muscle artifacts were identified semi-automatically (by applying individual z-value thresholds to the z-transformed sensor time-series, band-pass filtered between 110–140 Hz) and excluded. Second, eye-movement artifacts and cardiac signals were projected out of the data using independent component analysis. The 30-s stimulus blocks were then epoched into 1-s-long trials (4 reversals within one trial) and the 30-s rest blocks were also epoched into 1-s trials.

**Fig. 1:**
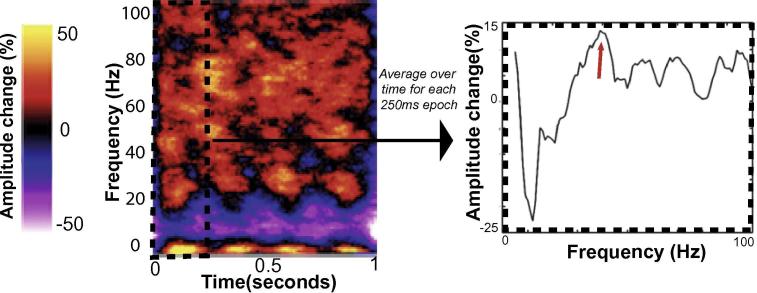
Method used to characterize the peak gamma power response, for each participant. After performing the Hilbert transform on each trail, and averaging over trials, we obtain power changes in the time–frequency domain, relative to baseline. Shown on the left is a time–frequency plot for one participant, at one visual contrast (50%). For each 250-ms epochs (corresponding to one checkerboard reversal), we average over time. We then extract the peak amplitude change from baseline (on the right) which is shown in this example to be approximately 15%, at 40 Hz (indicated by the red arrow). We take an average of the four values (one from each of the four 250-ms epochs) to give the final peak gamma power change from baseline.

For source localization, each participant’s anatomical MRI was divided into an irregular grid by warping the individual MRI to the MNI template brain and then applying the inverse transformation matrix to the regular MNI template grid (5-mm isotropic voxel resolution), allowing source estimates at brain locations directly comparable across participants. For each grid location inside the brain, the forward model (i.e., the lead field) was calculated for a single-dipole orientation by singular value decomposition, using a single-shell volume conduction model (Nolte, 2003). Source power at each location was estimated using an LCMV (linearly constrained minimum variance) beamformer, where the weights were computed using a covariance matrix calculated after band-pass filtering the data between 30 and 80 Hz, combining trials from all conditions. For each participant, the voxel of greatest increase in gamma power (30–80 Hz) was located within either the Calcarine sulcus (primary visual cortex) or two adjacent regions (cuneus and lingual gyrus), found by contrasting the 1-s stimulus epochs with the 1-s baseline epochs (as a percentage change from baseline). Anatomical masks were created using the AAL atlas (Tzourio-Mazoyer et al., 2002). At this peak location, the source-level time-series were reconstructed by multiplying the sensor-level data by the beamformer weights. Trials were represented in the time–frequency domain by calculating the amplitude envelope of analytic signal obtained with the Hilbert transform. Stimulus-induced peak gamma power was extracted, separately for each visual contrast condition, using the approach described in Fig. 1. This analysis was performed separately for the left and right eye acquisitions.

To assess potential alterations in transmission to the visual cortex, latencies of visual-evoked fields (VEFs) were characterized, for the left and right eyes separately, and across all five contrast conditions together. The trials were first re-epoched around the time of reversal (0 s), with the baseline period defined as -0.04 to 0 s, and the stimulus period as 0 to 0.21 s. The data were then low-pass filtered at 15 Hz and baseline corrected. The VEFs were first investigated at the sensor level (mirroring methods used clinically for VEPs) by averaging over trials for five posterior occipital sensors. The VEFs were subsequently also characterized in source space (more comparable with the analysis of the gamma power changes) by multiplying the pre-processed sensor-level data with the beamformer weights for the location found to be the peak gamma response to the checkerboard stimuli. For both types of analysis, the latency of the peak amplitude between 0 and 0.21 s was then extracted for each participant.

### MRI data analysis

#### Lesion filling

Lesion filling was carried out with the FSL function *lesion_filling*, following the protocol of Battaglini et al., 2012*,* to improve registration, segmentation and volumetric measures of brain tissue. In brief, a lesion mask was manually created by drawing around any visible lesions on the patient's T1-weighted image. At least one lesion was visible for 11 out of the 14 patients. For these 11 patients, the lesion mask, their T1-weighted image, and a white matter mask (FAST segmentation) was used in order to “fill” the lesion area in the T1-weighted image with intensities that are similar to those in the non-lesioned neighborhood (white matter only).

#### Tissue volumes

Brain tissue volume, normalized for subject head size, was estimated with SIENAX in FSL (Smith et al., 2001, Smith et al., 2002). For patients with visible lesions, their T1-weighted images with filled lesions were inputted. Brain and skull images were extracted from the single whole-head input data (Smith, 2002). The brain image was then affine-registered to MNI152 space (Jenkinson and Smith, 2001, Jenkinson et al., 2002), using the skull image to determine the registration scaling, and to obtain the volumetric scaling factor. Next, tissue-type segmentation with partial volume estimation was carried out (Zhang et al., 2001) in order to calculate total volume of brain tissue (and volume of gray and white matter separately), normalized for head size using the volumetric scaling factor.

Regional GM tissue volumes from the visual cortex were calculated for each subject. These visual ROIs were defined functionally, based on significant group activation to the visual checkerboard stimulus (explained below). The group visual ROI for the left and right eye stimulation was transformed from standard space to T1 subject space and masked with the GM partial volume image to give visual GM ROIs for each subject. Estimates of volume within these ROIs were then normalized with the volumetric scaling factor outputted from the SIENAX analysis.

#### BOLD and CBF response to the visual checkerboard stimulus

The BOLD signal was isolated by surround averaging the second echo to remove the tag-control signal, as described previously in Liu and Wong (2005). Registration of functional data to individual T1 structural data (linear, 6 degrees of freedom) and then to MNI standard space (linear, 12 degrees of freedom) was carried out using FSL FLIRT (Jenkinson and Smith, 2001, Jenkinson et al., 2012). Motion correction of time series data was performed using MCFLIRT (Jenkinson et al., 2002), with non-brain removal using BET (Smith et al., 2002b), spatial smoothing using a Gaussian kernel of FWHM 5 mm, with a high-pass temporal filter applied with a cut off of 90 s. The time series analyses were carried out using FEAT Version 6, part of FSL (Jenkinson et al., 2012). Using FEAT, perfusion time courses were modeled from the first echo data with the inclusion of regressors explicitly describing the tag-control signal differences. Five stimulus conditions, and an average across the conditions, were specified as six output contrasts relative to the rest conditions.

A high-level analysis was performed with FEAT using a mixed effects model (FLAME 1 + 2) to model the effect of group membership. *Z* statistic images were thresholded using clusters determined by *Z* > 2.3 and a (corrected) cluster significance threshold of *p* = 0.05 (Worsley, 2001). A group region of interest (ROI) was generated from the output of this group analyses. The group ROI consisted of common significant voxels (based on the thresholded z-statistic images, for the contrast averaged across conditions) among BOLD activity in the control group, BOLD activity in the patient group, CBF activity in the control group and CBF activity in the patient group. After binarizing this group ROI and transforming to subject space for each participant, a percentage signal increase in BOLD and percentage increase in CBF were calculated for each participant within that region. This higher-level analysis and group ROI creation were done separately for the left and right eye acquisitions. The final BOLD and CBF values were then averaged across eyes for each participant.

#### Baseline perfusion

As patients with MS are reported to be hypoperfused at rest (Swank et al., 1982, Brooks et al., 1984, Lycke et al., 1993, Sun et al., 1998, Law et al., 2004, Adhya et al., 2006, D’haeseleer et al., 2013) a measure of resting CBF in ml/per/100 g per min was quantified to establish if there were any differences in baseline perfusion. Baseline perfusion was estimated following a protocol described by Warnert et al. (2014), using in-house scripts that used AFNI and FSL-BASIL. In brief, ASL scans were first motion corrected using AFNI. All TIs (from both scans) were merged into one 4D dataset which included a single mean difference image per TI, averaged over the 16 volumes. The M_0_ image was registered to this perfusion series and a mask of the lateral ventricles was created, and this was used in the subsequent model to calculate the equilibrium magnetization of blood (*M*_0_). A two-compartment kinetic model was fitted to the multi-inversion time data to calculate baseline perfusion, in native space, in ml/100 g/min along with mean arrival time (Chappell et al., 2010). Individual subject GM masks (from partial-volume tissue-segmentation, see *Tissue Volumes*) were transformed to native space in order to estimate the baseline blood flow over GM. The standard-space ROI used in the checkerboard analysis was also transformed to native space, and the baseline perfusion in this region was used to convert fractional estimates of task-induced change in blood flow to changes in absolute blood flow units.

#### Characterizing neurovascular coupling

We characterized neurovascular coupling by fitting a linear model that reflected the relationship between the electrophysiological response and the hemodynamic response to the visual checkerboard stimulus. Three coupling models were fitted for each subject: the relationship between gamma oscillations and the relative BOLD signal, the relative CBF signal, and the quantified CBF signal. We used BOLD signal changes, dependent on both metabolism and flow, as this has been the focus of most previous studies relating MEG and fMRI signals. We used CBF signal changes as they reflect direct perfusion to the capillary bed, more localized to the active tissue (Buxton, 2009). We also quantified this CBF signal change in ml/100 g/min, due to evidence showing baseline CBF can affect BOLD and CBF responses to stimulus (e.g., Cohen et al., 2002), and that absolute changes in CBF may more closely represent the neuronal response to a stimulus (Whittaker et al., 2016).

For each subject, there were 10 data points: one point for each visual contrast and for each eye. We chose not to average across eyes in order to retain useful variance in the responses between eyes, therefore helping us to better model the relationship between MEG and fMRI signals. The gradient of the line, extracted for each participant, was taken to be our coupling measure, indicating the strength of the relationship between these signals.

#### Statistical analysis of group differences and stimulus responses

Statistical analysis was carried out using IBM SPSS Statistics (Version 20) and R software packages (R Core Team, 2016). Independent *t*-tests assessed differences between MS patients and controls on age, behavioral measures, tissue volumes and baseline signals. Mann–Whitney *U*-tests were used to assess differences between MS patients and controls on the visual acuity scores, for each contrast level tested. Mixed ANOVAs were used to assess the effect of group membership and eye on the latency of the peak (of the VEFs), and the effect of group membership and contrast level on peak gamma power, BOLD and CBF metrics. For the neurovascular coupling measure, gradients and intercepts were extracted from the linear model that was fitted separately for each person, and Mann–Whitney *U*-tests were used to test the differences in medians between MS patients and controls.

For the Mann–Whitney *U*-tests, an exact sampling distribution was used for *U* (Dineen and Blakesley, 1973). For each comparison, the shape of the distribution was similar between groups, as assessed by visual inspection, so medians were compared. GG in the results refers to the Greenhouse–Geisser correction used when the assumption of homogeneity of variances is violated. In these statistical analyses, all hypothesis testing was two-tailed. The family-wise error rate was controlled with the Holm–Bonferroni correction, a popular variant of the Bonferroni correction that is less conservative (Holm, 1979).

## Results

### Demographics and clinical profile

The demographic and clinical characteristics of the 14 patients and 10 healthy controls are reported in Table 1. Patients were significantly slower than controls when completing the 9-HPT task and showed a trend toward being significantly slower in the T25-FW task.

**Table 1:** Demographic and clinical characteristics for patients (*n* = 14) and controls (*n* = 10). Values are reported as Mean ± SEM. The 9-HPT is a mean of two trails for each hand. The T25-FW is a mean of two trials. One patient did not complete the T25-FW testing. The normalized brain volume is based on the patient's lesion-filled T1 weighted images. Significance is tested using two-tailed unpaired *t*-tests, except for differences in sex, which was tested with Fisher's Exact Test

	Patients	Controls	*P*-value
Age	43.46 ± 3.50	42.40 ± 3.73	0.69
Sex (M/F)	5/9	1/9	0.34
Disease duration (Years)	7.31 ± 2.06	–	–
EDSS (Median, Range)	3.0, 0–4.5	–	–
History of optic neuritis	6/14	–	–
9-HPT (s)	25.65 ± 0.82	22.41 ± 0.95	0.02
T25-FW (s)	13.08 ± 1.58	9.63 ± 0.19	0.05
PASAT-3s (No. correct responses)	46.54 ± 2.57	48.10 ± 3.37	0.71
PASAT-2s (No. correct responses)	31.92 ± 1.86	36.20 ± 2.95	0.21
Normalized Brain Volume (mm^3^)	1,492,761.57 ± 26644.57	1,523,284.20 ± 25006.90	0.43

### Visual acuity and VEFs

One patient was not included in the right eye group analysis due to blindness of the right eye. The log(MAR) values were extremely non-normal in their distribution across eyes and groups, and different cells of the design (group vs. eye vs. contrast level) had different variances. Therefore, as the group difference was the focus, separate Mann–Whitney *U* tests were used to test the group difference in the visual acuity at each contrast level, and the p values were corrected for multiple comparisons with the Holm–Bonferroni correction. Group differences were assessed at each contrast (100%, 25%, 10%, 5%, 2.5%, 1.25%, 0.6%) and for each eye (left, right). There were no significant group differences between MS patients and controls in visual acuity scores (Table 2).

**Table 2:** Median log(MAR) visual acuity scores, compared between MS patients (*n* = 12) and controls (*n* = 10), for each contrast level and eye. Higher median scores indicate greater visual acuity loss. Mann–Whitney *U*-tests were performed for each group comparison, and the test statistic and corresponding p-value is reported here. Using the Holm–Bonferroni correction, the last row shows the threshold at which that p-value is significant. There were no significant group differences between median visual acuity scores for any comparison

Eye	Visual Contrast (%)	Median		Mann–Whitney *U*	*P* value	Significant if less than
		Controls	Patients			
Left	100	0.10	0.00	52.5	0.63	0.016
	25	0.10	0.10	62	0.92	0.05
	10	0.20	0.20	69	0.58	0.01
	5	0.40	0.49	72.5	0.38	0.006
	2.50	0.85	1.00	80	0.20	0.004
	1.25	1.65	2.00	73	0.42	0.006
	0.60	2.00	2.00	69	0.58	0.01

Right	100	0.00	0.05	79.5	0.20	0.004
	25	0.10	0.10	79	0.23	0.005
	10	0.20	0.25	71	0.50	0.007
	5	0.40	0.49	75	0.35	0.005
	2.50	0.80	1.30	82.5	0.14	0.004
	1.25	2.00	2.00	71	0.47	0.007
	0.60	2.00	2.00	66	0.72	0.025

We investigated the effect of group (controls, patient) and eye (left, right) on the latency of the peak amplitudes of the VEFs, for the sensor and source space analyses. The data were normally distributed, with no outliers. Results reported here are Mean ± Standard Error of the Mean(SEM), expressed in milliseconds. For latencies calculated in sensor space, MS patients (146 ± 9) and controls (147 ± 10) did not have significantly different latencies (*F*(1,21) = 0.001, *p* = 0.98), and the left eye (156 ± 9) and the right eye (137 ± 8) also did not differ (*F*(1,21) = 3.04, *p* = 0.10), for both groups. The results were similar for latencies calculated in source space: there was no main effect of group (MS patients: 137 ± 5, controls: 142 ± 6, *F*(1,21) = 0.38, *p* = 0.55) and no main effect of eye (left: 145 ± 6, right: 134 ± 6, *F*(1,21) = 1.83, *p* = 0.19). There was no significant interaction between group and eye for the latencies calculated in sensor space, (*F*(1,21) = 0.001, *p* = 0.98), or source space (*F*(1,21) = 0.16, *p* = 0.70).

### MEG and fMRI responses to reversing checkerboard stimuli

There were no significant differences in GM volume, baseline CBF or baseline gamma power between MS patients and controls, across the whole brain and within the regions of interest used to characterize the visual response to the checkerboard stimulus (Table 3).

**Table 3:** Tissue volumes, and baseline signals compared between groups. GM tissue volumes and baseline cerebral blood flow (bCBF) are compared for the total GM, as well as for GM within the left ROI (L) and the right (R) which was used to extract the BOLD and CBF responses to the visual checkerboard stimulus. Baseline gamma power is a mean of the power at each frequency between 30 and 80 Hz, averaged over the 30-s rest block, but extracted from the same location as the peak gamma response to the checkerboard stimuli. The *p*-values for GM Volume (3 comparisons), for bCBF (3 comparisons) and bGamma Power (2 comparisons) were corrected separately and compared against the Holm–Bonferroni corrected thresholds: a is significant at *p* < 0.017, b at *p* < 0.05, c at *p* < 0.025

	Group	N	Mean	Std. Dev	*P* value
GM Volume (Total)	Controls	10	788328.70	55506.80	0.29^a^
(mm^3^)	Patients	14	755677.88	66346.84	
GM Volume (L)	Controls	10	5514.29	673.20	1.00^c^
(mm^3^)	Patients	14	5515.19	768.02	
GM Volume (R)	Controls	10	4154.44	530.05	0.90^b^
(mm^3^)	Patients	14	4184.46	585.56	
bCBF GM (Total)	Controls	10	36.91	10.17	0.90^c^
(ml/100 g/min)	Patients	13	37.34	6.32	
bCBF GM (L)	Controls	9	57.73	21.18	0.45^a^
(ml/100 g/min)	Patients	13	51.96	15.16	
bCBF GM (R)	Controls	9	57.30	21.00	0.48^b^
(ml/100 g/min)	Patients	13	51.70	16.81	
bGamma Power (L)	Controls	10	6.10	0.83	0.10^b^
(*10^−14^ Tesla)	Patients	13	7.07	1.63	
bGamma Power (R)	Controls	10	6.80	1.19	0.94^c^
(*10^−14^ Tesla)	Patients	13	6.80	1.29	

### Spatial comparison of MEG and fMRI results

**Fig. 2:**
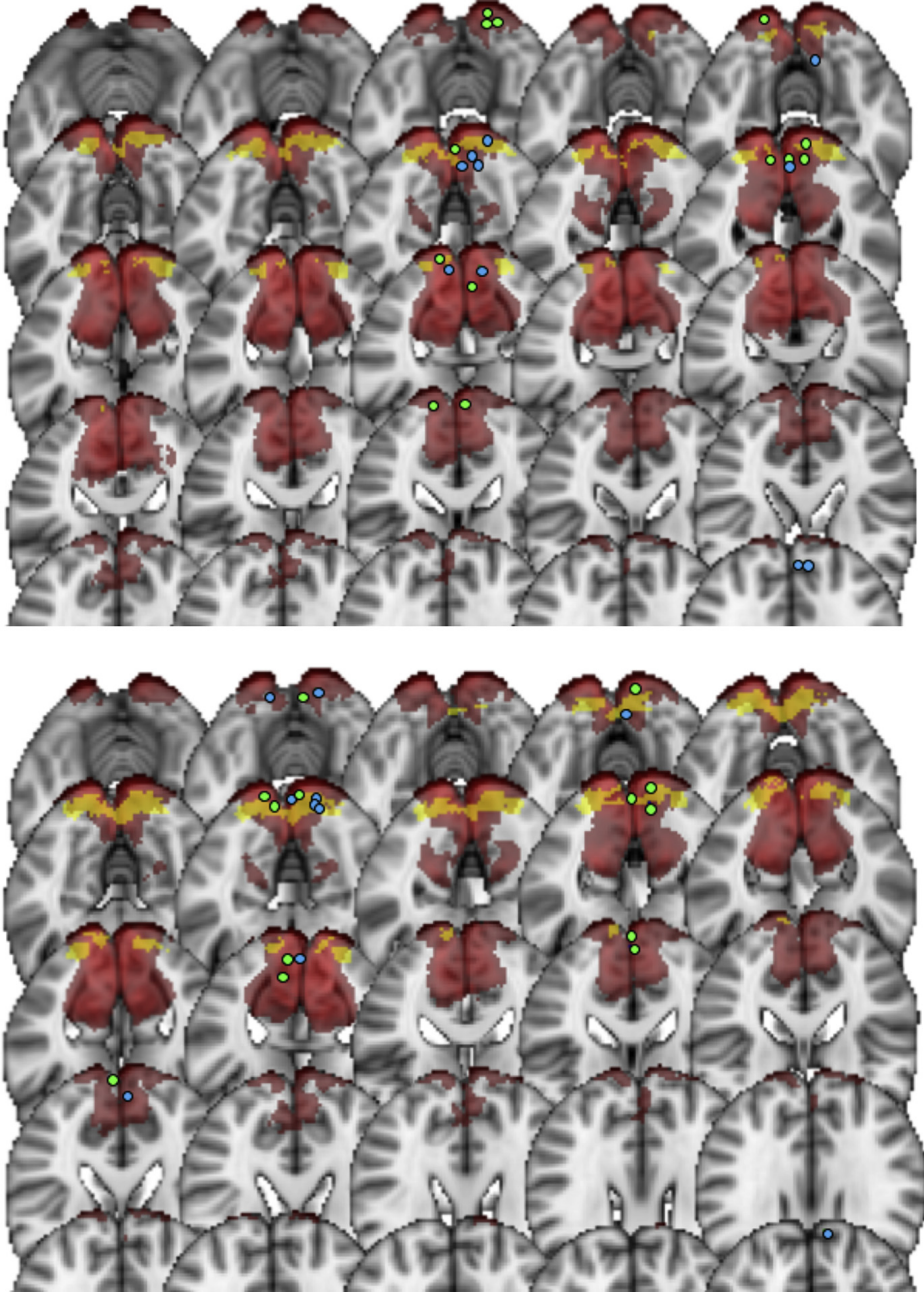
The location of the ROI used to extract the BOLD and CBF responses (yellow) for every participant, overlaid on the primary visual cortex (red). The top plot shows the left eye analysis and the bottom plot shows the right eye analysis. The dots indicate the location of the peak voxel (peak of the gamma response, percentage change from baseline) for each individual participant (blue = controls, green = MS patients), which was used for the time–frequency analysis of the MEG data. This is shown for *n* = 10 controls *n* = 13 MS patients.

**Fig. 3:**
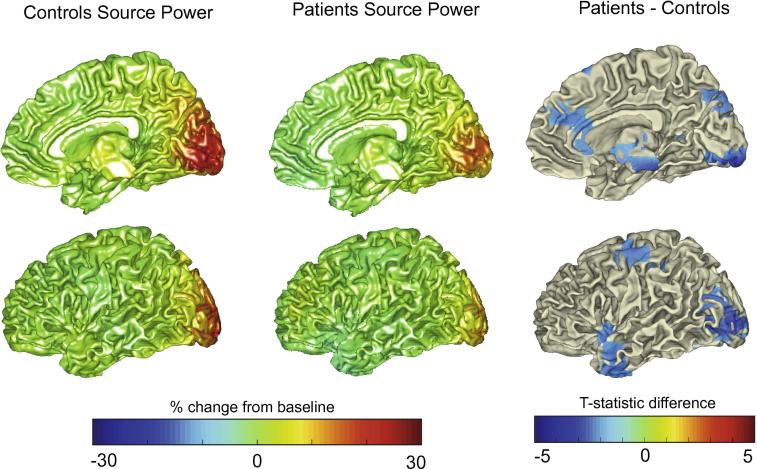
Beamformer contrast images (band-pass filtered 30–80 Hz) measured as percentage change between stimulus and baseline, projected onto a template brain surface. This is shown for controls (left column) and MS patients (middle column). The right column illustrates the *t*-statistic values for the difference between patients and controls (negative values indicating lower amplitude for patients than controls). Simply for illustration purposes, *t*-values are plotted here at the uncorrected level. The data were averaged over both eyes. Only right medial and lateral views are shown; the same trends were seen for left.

**Fig. 4:**
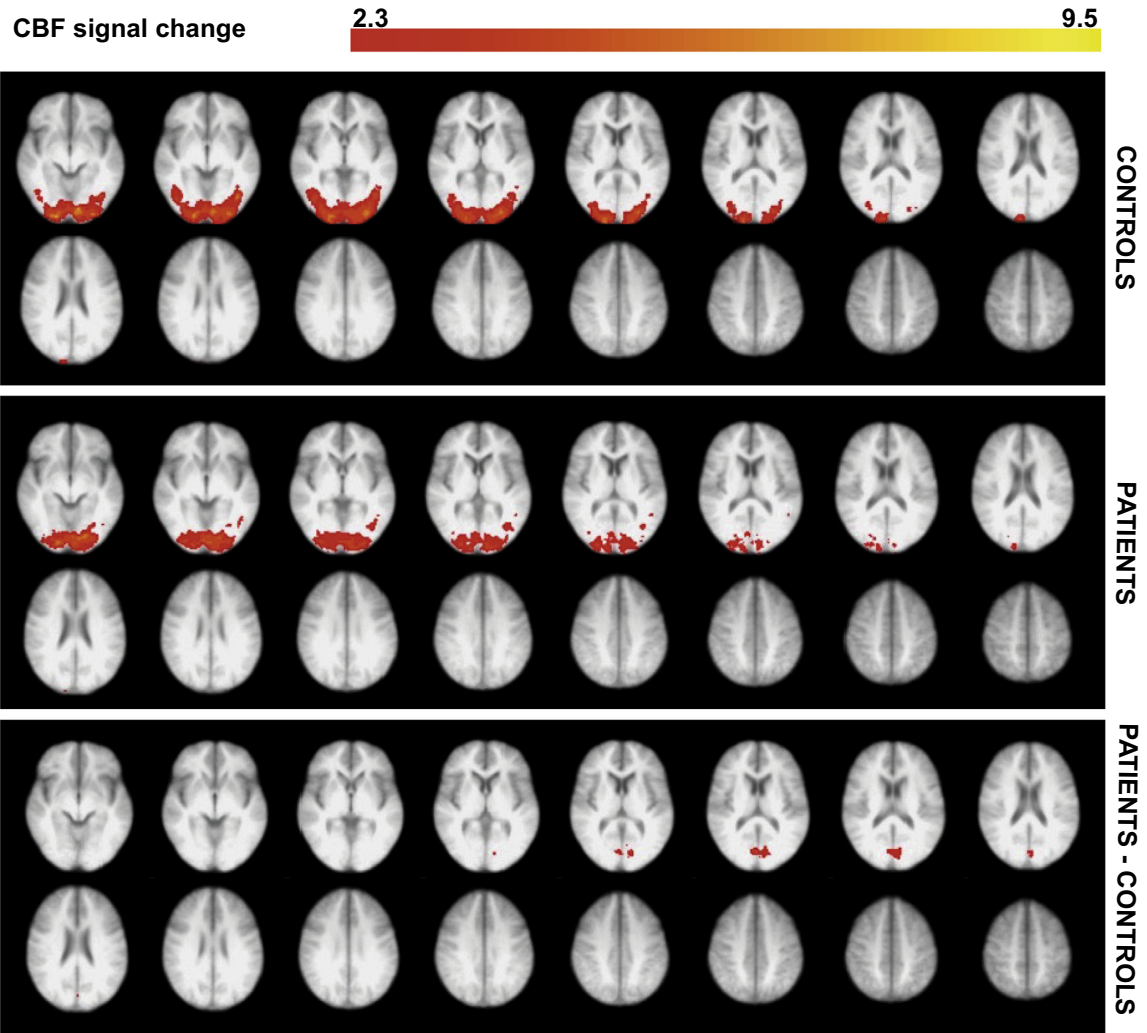
Significant CBF voxels at the group level in response to the visual checkerboard stimulus, compared between MS patients and controls. These data are an average of both eyes. The significant activity shown is the average activity across all visual stimulus conditions. Voxels were thresholded using clusters determined by *Z* > 2.3 and a (corrected) cluster significance threshold of *p* = 0.05 (Worsley, 2001). The bottom plot shows voxels that showed significantly greater activity in the patient group, compared with controls. This activity was localized to the intracalcarine and supracalcarine cortex, as well as the cuneus (using Harvard-Oxford Cortical Structural Atlas). There were no voxels showing significantly greater activity for the control group compared to the MS patients. The right side of the image corresponds to the left side of the brain.

**Fig. 5:**
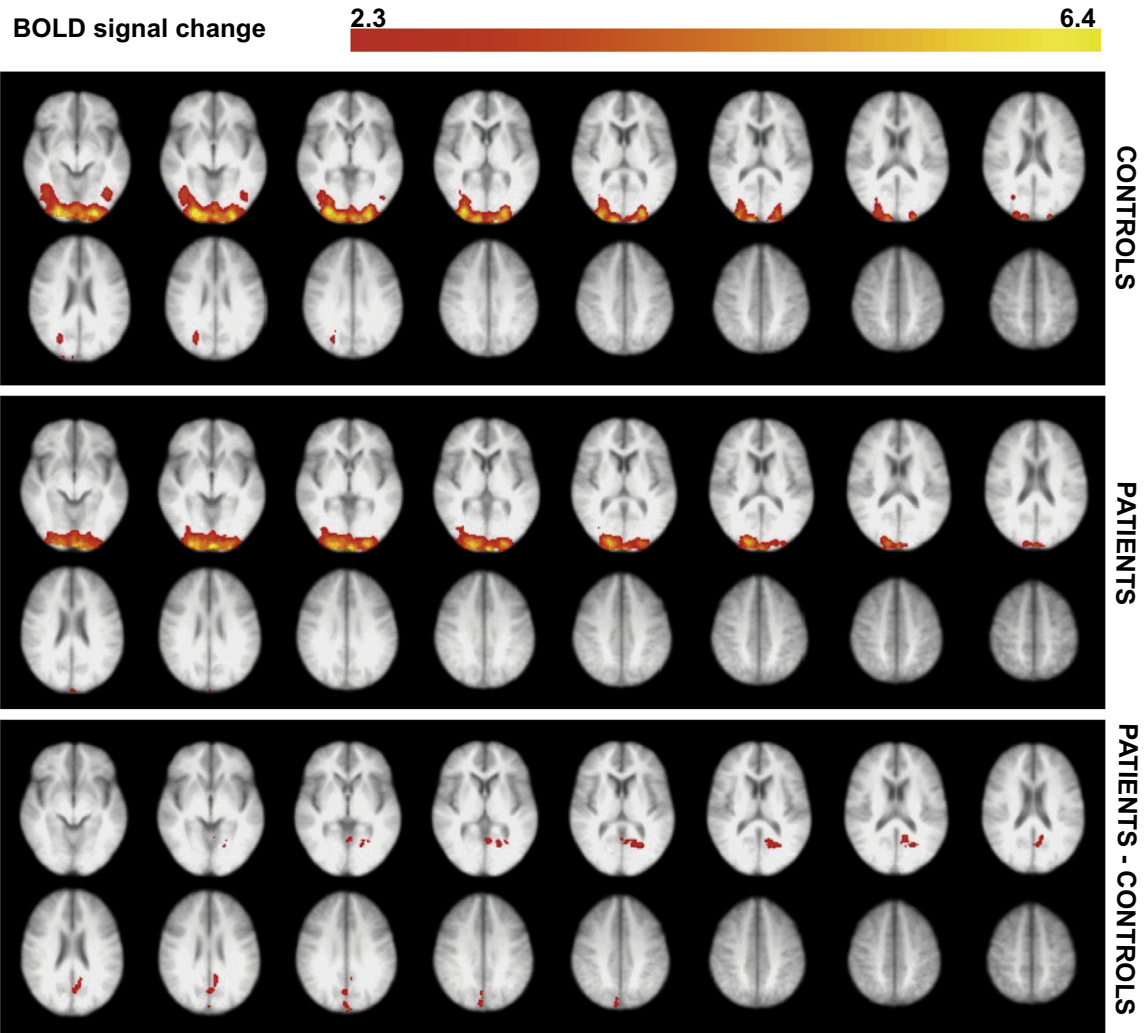
Significant BOLD voxels at the group level in the visual checkerboard stimulus, compared between MS patients and controls. These data are an average of both eyes. The significant activity shown is the average activity across all visual stimulus conditions. Voxels were thresholded using clusters determined by *Z* > 2.3 and a (corrected) cluster significance threshold of *p* = 0.05 (Worsley, 2001). The bottom plot shows voxels that showed significantly greater activity in the patient group, compared with controls. This activity was localized to the lingual gyrus, intracalcarine cortex, pre-cuneus and cuneus (using Harvard-Oxford Cortical Structural Atlas). There were no voxels showing significantly greater activity for the control group compared to the MS patients. The right side of the image corresponds to the left side of the brain.

One control’s fMRI data were not useable due a corrupted image file. Fig. 2 displays the location of the ROI used in the BOLD and CBF analyses, for all participants, overlaid onto the primary visual cortex. Included in Fig. 2 are the locations of the peak gamma responses for each patient and control, where the time–frequency analysis was performed. Fig. 3 shows the whole-brain MEG source localization plots for each group. Fig. 4 shows the whole-brain CBF activity for each group, and Fig. 5 the whole-brain BOLD activity.

### Effect of group and stimulus contrast on BOLD, CBF and gamma power change

**Fig. 6:**
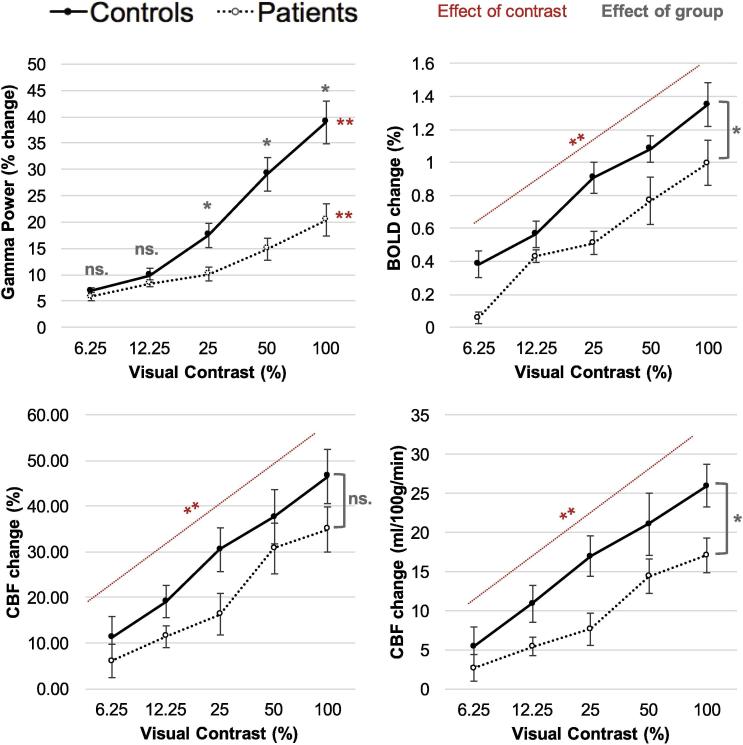
The effect of group and visual contrast on peak gamma power, BOLD signal, CBF signal (percentage change from baseline) and CBF signal (ml/100 g/min change from baseline)*.* The graph displays Mean ± SEM. For BOLD, CBF and CBF quantified the effect of group was not significantly different across contrast level, so the p values refer to the main effect. For Gamma, there was an interaction between group and contrast level so the p values refer to the simple main effects. ^*^p < 0.05, ^**^p < 0.001. All pairwise comparisons between contrast levels were significant at an alpha level of 0.05, with Holm–Bonferroni correction.

Given the absence of significant differences in the visual acuity scores and latency of the peak amplitudes between groups, the effect of group and stimulus contrast was statistically tested on data averaged across the two eyes. Fig. 6 displays the effect of group and stimulus contrast on BOLD, CBF and peak gamma power signal changes.

BOLD, CBF quantified and CBF percent signals increased significantly as stimulus contrast increased: *F (1.75,33.17*) = *46.59, p < 0.001 GG; F (4,76*) = *35.41, p < 0.001*; *F (4,76*) = *35.63, p < 0.001,* respectively. MS patients had significantly lower signal responses, compare with controls, for BOLD and CBF quantified signals, but this was not significant for CBF percent: *F (1,19*) = *7.97, p = 0.01*; *F (1,19*) = *6.62, p = 0.02*; *F (1,19*) = *2.76, p = 0.11*, respectively. All pairwise comparisons between contrast levels were significant. There was no significant interaction between the effect of contrast and group on BOLD changes (*F (1.75, 33.17*) = *0.86, p = 0.42, GG*) CBF quantified changes (*F (4, 76*) = *1.25, p = 0.30)*, or CBF percent changes (*F (4, 76*) = *0.70, p = 0.59*).

For the MEG results, there was a significant interaction between the effect of contrast and group on the peak gamma power changes (*F (1.50,31.54*) = *7.87, p = 0.01, GG*). Therefore, simple main effects were investigated. There was no significant group difference at the 6.25% or 12.5% contrast levels (*F (1,21*) = *1.13, p = 0.30*; *F (1,21*) = *1.99, p = 0.17*, respectively), but MS patients showed significantly lower peak gamma power changes at 25%, 50%, and 100% (*F (1,21*) = *6.28, p = 0.02*; *F (1,21*) = *12.13, p < 0.01*; *F (1,21*) = *10.71, p < 0.01,* respectively). Peak gamma power signals increased significantly as stimulus contrast increased, for both the control group (*F (1.59,14.29*) = *46.03, p < 0.001, GG*) and the patient group (*F (1.12, 15.99*) = *22.46, p < 0.001*, *GG*). All pairwise comparisons between contrast levels were significant, except between 6.25% and 12.5%, and 12.5% and 25% in the patient group.

### Neurovascular coupling in MS patients and controls

**Fig. 7:**
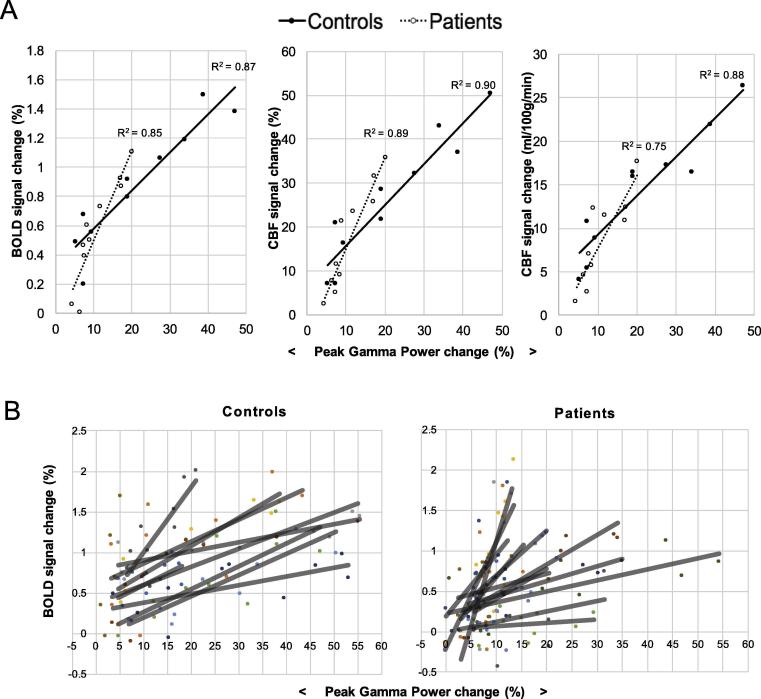
(A) shows the relationship between peak gamma power change and BOLD, CBF and CBF changes (quantified) in response to the visual checkerboard stimulus. Group median values are plotted. Each point represents a different contrast level for each eye. The reduced range of electrophysiological and the hemodynamic responses are evident in the patient group. (B) The relationship between peak gamma power change and BOLD change shown for each control (*n* = 9) and each patient (*n* = 12) separately. The different colors represent the different participants and the black lines the linear model fit.

The relationship between peak gamma power and BOLD and CBF signals was compared between groups. Fig. 7 visually displays these coupling relationships, and Table 4 shows the statistical testing between groups.

**Table 4:** Median gradients and intercepts compared between MS patients and controls, using peak gamma power to predict BOLD, CBF and CBF quantified (CBFq). Mann–Whitney *U*-tests were performed for each group comparison, and the test statistic and corresponding p-value is reported here. The *p* values are compared against Holm–Bonferroni corrected alpha levels, corrected for two dependent tests (gradient and intercepts)

Outcome measure		Median		Mann–Whitney *U*	*P* value	Significant if less than:
		Controls	Patients			
Gradient	BOLD	0.03	0.04	66	0.42	0.050
	CBF	1.03	1.30	65	0.46	0.050
	CBF(q)	0.67	0.58	58	0.81	0.050

Intercept	BOLD	0.31	0.04	24	0.03	0.025
	CBF	5.08	1.97	38	0.28	0.025
	CBF(q)	3.91	1.16	35	0.19	0.025

Fig. 7A displays the coupling relationships using the median values across each group. At this group averaged level, there is a good fit between the MEG and fMRI signals. The MS patients appear to have higher gradients on average (i.e., for the same peak gamma power change, higher BOLD and CBF change), as well as lower intercepts on average. Fig. 7B shows what the coupling relationship looks like for every participant separately, showing that the patient group displays more variability in the coupling between peak gamma power and BOLD change compared to the controls. CBF coupling results for each participant (not shown) showed similar trends.

To statistically test these coupling differences, the median gradients and intercepts were extracted for each person and compared between groups. In general, higher gradients and lower intercepts are seen in the patient group, but no significant differences are found between groups (Table 4).

## Discussion

We investigated the neuronal and hemodynamic responses to a reversing checkerboard visual stimulus in relapsing–remitting MS, observing smaller peak gamma power changes and BOLD and CBF responses. While the range of electrophysiological and hemodynamic responses were altered in MS, we found no significant group difference in the coupling relationship between these responses, indicating that neurovascular coupling may remain intact in MS. While the lack of significant differences between groups may be due to the limited statistical power of this study, due to relatively small samples sizes, it may also reflect the complexity and heterogeneity of MS as a disease, and neurovascular coupling as a biological process.

### Source localization of gamma oscillations, BOLD and CBF

The group ROI used to extract the BOLD and CBF signals for all participants was located clearly in the primary visual cortex (Fig. 2). For characterizing the gamma response, we searched for the peak gamma power change within the calcarine sulcus and two adjacent regions: the cuneus and lingual gyrus. This was because previous studies suggest visual gamma, in response to this type of stimulus, to be located in the primary visual cortex. This method also ensured that the area was comparable to the fMRI source while allowing for some error in MEG signal localization. Although we took a ROI approach to characterizing the peak gamma, CBF and BOLD responses, we also visualized these responses across the whole brain. At the whole-brain group-averaged level, we saw a reduction in gamma power responses in visual areas for the MS patients (Fig. 3). A reduction was also seen in sub-cortical and temporal regions; this possibly indicates activity differences more extensively along visual processing pathways, yet this was not further explored. For the CBF and BOLD group-average plots, a similar pattern of activity in the early visual cortex was seen for both groups (Fig. 4, Fig. 5). There were voxels within the intra/supra-calcarine cortex and cuneus (for CBF) and the lingual gyrus, intracalcarine cortex, and cuneus (for BOLD) showing significantly greater activity in the MS group compared to the controls. A possible explanation of this result is compensatory functional reorganization of the cortex in MS patients (Werring et al., 2000, Tomassini et al., 2012).

### Reduction in peak visual gamma, BOLD and CBF responses in MS

Here we used the oscillatory activity in the gamma band (30–80 Hz) to characterize the neuronal response. Broadly, the gamma rhythm is theorized to reflect the balance between excitatory and inhibitory signaling; networks of fast-spiking, parvalbumin-expressing, GABAergic interneurons act on pyramidal cells to bring about synchronous inhibitory post synaptic potentials (Bartos et al., 2007, Cardin et al., 2009, Buzsáki and Wang, 2012). A large body of research shows an increase in gamma power when functional networks are engaged, widely across the brain and for many different processes (e.g., Jensen et al., 2007, Nyhus and Curran, 2010; reviewed by Jia and Kohn, 2011). The functional role of gamma oscillations is not fully known, but it is thought to be in attention, perception and mediation of information transfer across different cortical areas (Fries, 2009).

Disruptions in gamma oscillations have been reported in many brain disorders (Uhlhaas and Singer, 2006). In MS, task-induced changes have remained largely unexplored until recently (e.g., Barratt et al., 2017, Arpin et al., 2017). Barratt et al. (2017) reported significantly reduced visual gamma amplitudes in a similar MS population, the only other study, to our knowledge, that has investigated task-induced gamma oscillatory changes in MS. There is evidence that parvalbumin-expressing GABAergic interneurons, thought to contribute to gamma oscillations, are reduced in normal appearing GM of the motor cortex in MS (Clements et al., 2008), and that secondary progressive MS patients have significantly lower GABA levels in the hippocampus and sensorimotor cortex (Cawley et al., 2015). GABA concentration has also been related to visual gamma oscillations and BOLD signals in healthy subjects in the visual cortex (Muthukumaraswamy et al., 2009, Magazzini et al., 2016). Therefore, a possible interpretation of our results is that these gamma power reductions in the visual cortex of MS patients could be an indicator of early GM dysfunction, mediated by GABAergic changes. However, we did not see a significant reduction in GM visual cortices or in whole-brain volumes in the MS group when compared to the healthy control group. Although, again, this negative finding may result from a small sample size, a contributing factor may be ongoing inflammation, as participants were treatment naïve. The contribution of inflammation to an increase in brain volume (and thus to an apparent normalization despite MS pathology) has been indirectly demonstrated by showing that the onset of disease modifying treatment leads to the occurrence of brain volume reduction, a phenomenon called “pseudo-atrophy” (Gasperini et al., 2002, Zivadinov et al., 2008, Vidal-Jordana et al., 2016).

Although they presented with preserved visual acuity and latency of visual-evoked fields, the MS patients showed significant hemodynamic alterations, in the form of reduced BOLD and CBF responses to the visual checkerboard, in the primary visual cortex. In the context of largely preserved neurovascular coupling, the reduced hemodynamic response is consistent with the reduced electrophysiological response. Changes in the BOLD response to visuomotor tasks have been previously demonstrated, showing that inflammation and white matter structural damage play a role in altering hemodynamic responses in MS (Tomassini et al., 2016, Hubbard et al., 2016). Our alterations in BOLD and CBF responses support these findings, as well as altered responses to visual stimuli at different contrasts (Faro et al., 2002). Uzuner and Uzuner (2016), also using a 2-Hz reversing checkerboard stimulus, found blood flow velocities in the posterior cerebral arteries to be higher in a large MS patient group in the state of relapse. Though a different measure, this is in contrast to the reduced blood flow responses we reported in this MS group, but highlights the potential impact of testing MS participants at different stages of the disease.

### No significant alteration of neurovascular coupling in MS

The relationship between the peak gamma power change and the BOLD/CBF response (using the variance given by the visual contrast manipulation) was our empirical measure of neurovascular coupling. While this is intuitive, assuming that the blood flow response only reflects a coupling with gamma oscillatory activity is simplistic. The amplitude of gamma oscillations can be modulated by the phase of slower oscillations, termed cross-frequency phase-amplitude coupling (Buzsáki and Wang, 2012), and an increase in gamma power is often accompanied by a decrease in power of lower frequencies. BOLD and gamma oscillations are also known to be decoupled in some circumstances. For example, in the visual cortex, gamma amplitudes are altered with changes in the spatial frequency and color of the stimuli, but BOLD signals are not (Muthukumaraswamy and Singh, 2008, Muthukumaraswamy and Singh, 2009, Swettenham et al., 2013). Despite these limitations, and although the temporal relationship of MEG and fMRI signals is complex (Hall et al., 2014), they are generally thought to originate from the same electrophysiological source and have reasonable spatial overlap. Gamma oscillations have high test–retest reliability, with stable features within the same participants for at least 4 weeks (Muthukumaraswamy et al., 2010, Tan et al., 2016), which is important considering the practical limitation of doing the MEG and fMRI scanning sessions separately.

In this study, we could not demonstrate significant differences in neurovascular coupling between the MS patients and controls. While this may be related to the power of the study, it may also reflect the complexity of the biology underlying the relationship between neuronal activity and the hemodynamic response, which in MS is affected by the inflammatory *milieu*. Indeed, the response of blood vessels to neuronal activity is not only mediated by reactivity of the smooth muscle cells, but also by neuronal and glial signaling, involving many chemical mediators. Increased levels of both vasodilators (e.g., nitric oxide, Smith and Lassmann, 2002) and vasoconstrictors (e.g., endothelin-1, D’haeseleer et al., 2013) have been reported in MS, due to the proliferation of glial cells to damaged areas, which could interfere with neurovascular coupling pathways in contrasting ways. In line with the hypothesis that inflammation affects neurovascular coupling, there is the evidence that the MS group appeared to display more variance in their coupling relationships, suggesting a greater inter-individual variability.

While not significantly different from healthy volunteers, the analysis of the neurovascular coupling showed a trend for the MS group to have lower intercepts and higher gradients, when predicting the BOLD and CBF changes from the peak gamma power changes. An increased blood flow response, for same gamma power change, may seem counterintuitive considering the reports of blood vessels being less reactive in MS (Marshall et al., 2014, Marshall et al., 2016). However, an increased blood flow response could reflect the need to deliver more oxygen or nutrients to tissue, if there is inefficiency in their use to support a given level of electrophysiological activity.

We found evidence for reduced neuronal and hemodynamic responses in the early visual cortex in MS in response to visual stimulation, in the absence of substantial functional impairments to visual acuity or delayed visual-evoked fields. We could not demonstrate a significant alteration in neurovascular coupling in MS patients. Further research into neurovascular and metabolic function across the whole brain, and at different disease stages, will help uncover the importance of these processes in MS.

## References

[b0005] Adhya S., Johnson G., Herbert J., Jaggi H., Babb J.S., Grossman R.I., Inglese M. (2006). Pattern of hemodynamic impairment in multiple sclerosis: dynamic susceptibility contrast perfusion MR imaging at 3.0 T. Neuroimage.

[b0010] Arpin D.J., Heinrichs-Graham E., Gehringer J.E., Zabad R., Wilson T.W., Kurz M.J. (2017). Altered sensorimotor cortical oscillations in individuals with multiple sclerosis suggests a faulty internal model. Hum Brain Mapp.

[b0015] Attwell D., Iadecola C. (2002). The neural basis of functional brain imaging signals. Trends Neurosci.

[b0020] Attwell D., Buchan A.M., Charpak S., Lauritzen M., MacVicar B.A., Newman E.A. (2010). Glial and neuronal control of brain blood flow. Nature.

[b0025] Barratt E.L., Tewarie P.K., Clarke M.A., Hall E.L., Gowland P.A., Morris P.G., Francis S.T., Evangelou N., Brookes M.J. (2017). Abnormal task driven neural oscillations in multiple sclerosis: a visuomotor MEG study. Hum Brain Mapp.

[b0030] Bartos M., Vida I., Jonas P. (2007). Synaptic mechanisms of synchronized gamma oscillations in inhibitory interneuron networks. Nature Rev Neurosci.

[b0035] Battaglini M., Jenkinson M., De Stefano N. (2012). Evaluating and reducing the impact of white matter lesions on brain volume measurements. Hum Brain Mapp.

[b0040] Brooks D.J., Leenders K.L., Head G., Marshall J., Legg N.J., Jones T. (1984). Studies on regional cerebral oxygen utilisation and cognitive function in multiple sclerosis. J Neurol Neurosurg Psychiatry.

[b0045] Buxton R.B. (2009). Introduction to functional magnetic resonance imaging: principles and techniques.

[b0050] Buzsáki G., Wang X.J. (2012). Mechanisms of gamma oscillations. Annu Rev Neurosci.

[b0055] Cawley N., Solanky B.S., Muhlert N., Tur C., Edden R.A., Wheeler-Kingshott C.A., David H.M., Alan J.T., Ciccarelli O. (2015). Reduced gamma-aminobutyric acid concentration is associated with physical disability in progressive multiple sclerosis. Brain.

[b0060] Cantin S., Villien M., Moreaud O., Tropres I., Keignart S., Chipon E., Le Bas J.F., Warnking J., Krainik A. (2011). Impaired cerebral vasoreactivity to CO2 in Alzheimer's disease using BOLD fMRI. Neuroimage.

[b0065] Cardin J.A., Carlén M., Meletis K., Knoblich U., Zhang F., Deisseroth K., Tsai L.H., Moore C.I. (2009). Driving fast-spiking cells induces gamma rhythm and controls sensory responses. Nature.

[b0070] Chappell M.A., MacIntosh B.J., Donahue M.J., Günther M., Jezzard P., Woolrich M.W. (2010). Separation of macrovascular signal in multi-inversion time arterial spin labelling MRI. Magn Reson Med.

[b0075] Clements R.J., McDonough J., Freeman E.J. (2008). Distribution of parvalbumin and calretinin immunoreactive interneurons in motor cortex from multiple sclerosis post-mortem tissue. Exp Brain Res.

[b0080] Cohen E.R., Ugurbil K., Kim S.G. (2002). Effect of basal conditions on the magnitude and dynamics of the blood oxygenation level-dependent fMRI response. J Cereb Blood Flow Metab.

[b0085] Cover K.S., Vrenken H., Geurts J.J., van Oosten B.W., Jelles B., Polman C.H., Stam C.J., van Dijk B.W. (2006). Multiple sclerosis patients show a highly significant decrease in alpha band interhemispheric synchronization measured using MEG. Neuroimage.

[b0090] Cutter G.R., Baier M.L., Rudick R.A., Cookfair D.L., Fischer J.S., Petkau J., Syndulko K., Weinshenker B.G., Antel J.P., Confavreux C., Elison G.W. (1999). Development of a multiple sclerosis functional composite as a clinical trial outcome measure. Brain.

[b0095] Demanuele C., James C.J., Sonuga-Barke E.J. (2007). Distinguishing low frequency oscillations within the 1/f spectral behaviour of electromagnetic brain signals. Behav Brain Funct.

[b0100] D’haeseleer M., Beelen R., Fierens Y., Cambron M., Vanbinst A.M., Verborgh C., Demey J., DeKeyser J. (2013). Cerebral hypoperfusion in multiple sclerosis is reversible and mediated by endothelin-1. PNAS.

[b0105] Dineen L.C., Blakesley B.C. (1973). Algorithm AS 62: generator for the sampling distribution of the Mann-Whitney U statistic. J Appl Stat.

[b0115] Faro S.H., Mohamed F.B., Tracy J.I., Elfont R.M., Pinus A.B., Lublin F.D., Koenigsberg R.A., Chen C.Y., Tsai F.Y. (2002). Quantitative functional MR imaging of the visual cortex at 1.5 T as a function of luminance contrast in healthy volunteers and patients with multiple sclerosis. AJNR.

[b0120] Fries P. (2009). Neuronal gamma-band synchronization as a fundamental process in cortical computation. Annu Rev Neurosci.

[b0125] Gasperini C., Paolillo A., Giugni E., Galgani S., Bagnato F., Mainero C., Onesti E., Bastianello S., Pozzilli C. (2002). MRI brain volume changes in relapsing-remitting multiple sclerosis patients treated with interferon beta-1a. Mult Scler.

[b0130] Ge Y., Zhang Z., Lu H., Tang L., Jaggi H., Herbert J., Babb J.S., Rusinek H., Grossman R.I. (2012). Characterizing brain oxygen metabolism in patients with multiple sclerosis with T2-relaxation-under-spin-tagging MRI. J Cereb Blood Flow Metab.

[b0135] Girouard H., Iadecola C. (2006). Neurovascular coupling in the normal brain and in hypertension, stroke, and Alzheimer disease. J Appl Physiol.

[b0140] Goodyear B.G., Menon R.S. (1998). Effect of luminance contrast on BOLD fMRI response in human primary visual areas. J Neurophysiol.

[b0145] Hall S.D., Holliday I.E., Hillebrand A., Singh K.D., Furlong P.L., Hadjipapas A., Barnes G.R. (2005). The missing link: analogous human and primate cortical gamma oscillations. Neuroimage.

[b0150] Hall E.L., Robson S.E., Morris P.G., Brookes M.J. (2014). The relationship between MEG and fMRI neuroimage.

[b0155] Hansen P.H., Kringelbach M., Salmelin R. (2010). MEG: an introduction to methods.

[b0160] Henrie J.A., Shapley R. (2005). LFP power spectra in V1 cortex: the graded effect of stimulus contrast. J Neurophysiol.

[b0170] Holm S. (1979). A simple sequentially rejective multiple test procedure. Scand J Stat.

[b0180] Hubbard N.A., Turner M., Hutchison J.L., Ouyand A., Strain J., Oasay L., Sundaram S., Davis S., Remington G., Brigante R., Huang H., Hart J., Frohman T., Frohman E., Biswal B.B., Rypma B. (2016). Multiple sclerosis-related white matter microstructural change alters the BOLD hemodynamic response. J Cereb Blood Flow Metab.

[b0190] Iadecola C. (2004). Neurovascular regulation in the normal brain and in Alzheimer's disease. Nat Rev Neurosci.

[b0195] Jia X., Kohn A. (2011). Gamma rhythms in the brain. PLoS Biol.

[b0200] Jenkinson M., Smith S. (2001). A global optimisation method for robust affine registration of brain images. Med Image Anal.

[b0205] Jenkinson M., Bannister P., Brady M., Smith S. (2002). Improved optimization for the robust and accurate linear registration and motion correction of brain images. Neuroimage.

[b0210] Jenkinson M., Beckmann C.F., Behrens T.E., Woolrich M.W., Smith S.M. (2012). Fsl Neuroimage.

[b0215] Jensen O., Kaiser J., Lachaux J.P. (2007). Human gamma-frequency oscillations associated with attention and memory. Trends Neurosci.

[b0225] Kleiner M., Brainard D., Pelli D., Ingling A., Murray R., Broussard C. (2007). What’s new in Psychtoolbox-3. Perception.

[b0230] Kurtzke J.F. (1983). Rating neurologic impairment in multiple sclerosis: an expanded disability status scale (EDSS). Neurology.

[b0235] Law M., Saindane A.M., Ge Y., Babb J.S., Johnson G., Mannon L.J., Herbet J., Grossman R.I. (2004). Microvascular abnormality in relapsing-remitting multiple sclerosis: perfusion MR imaging findings in normal-appearing white matter 1. Radiology.

[b0240] Liu T.T., Wong E.C. (2005). A signal processing model for arterial spin labeling functional MRI. Neuroimage.

[b0245] Logothetis N.K. (2002). The neural basis of the blood–oxygen–level–dependent functional magnetic resonance imaging signal. Philos Trans R Soc Lond B Biol Sci.

[b0250] Lycke J., Wikkelsö C., Bergh A.C., Jacobsson L., Andersen O. (1993). Regional cerebral blood flow in multiple sclerosis measured by single photon emission tomography with technetium-99m hexamethyl-propyleneamine oxime. Eur Neurol.

[b0255] Magazzini L., Muthukumaraswamy S.D., Campbell A.E., Hamandi K., Lingford-Hughes A., Myers J.F.M., Nutt D.J., Sumner P.S., Wilson S.J., Singh K.D. (2016). Significant reductions in human visual gamma frequency by the gaba reuptake inhibitor tiagabine revealed by robust peak frequency estimation. Hum Brain Mapp.

[b0260] Marshall O., Lu H., Brisset J.C., Xu F., Liu P., Herbert J., Grossman R.I., Ge Y. (2014). Impaired cerebrovascular reactivity in multiple sclerosis. JAMA Neurol.

[b0265] Marshall O., Chawla S., Lu H., Pape L., Ge Y. (2016). Cerebral blood flow modulation insufficiency in brain networks in multiple sclerosis: a hypercapnia MRI study. J Cereb Blood Flow Metab.

[b0270] Metea M.R., Newman E.A. (2006). Glial cells dilate and constrict blood vessels: a mechanism of neurovascular coupling. J Neurosci.

[b0275] Mukamel R., Gelbard H., Arieli A., Hasson U., Fried I., Malach R. (2005). Coupling between neuronal firing, field potentials, and FMRI in human auditory cortex. Science.

[b0280] Muthukumaraswamy S.D., Singh K.D. (2008). Spatiotemporal frequency tuning of BOLD and gamma band MEG responses compared in primary visual cortex. Neuroimage.

[b0285] Muthukumaraswamy S.D., Edden R.A., Jones D.K., Swettenham J.B., Singh K.D. (2009). Resting GABA concentration predicts peak gamma frequency and fMRI amplitude in response to visual stimulation in humans. PNAS.

[b0290] Muthukumaraswamy S.D., Singh K.D. (2009). Functional decoupling of BOLD and gamma-band amplitudes in human primary visual cortex. Hum Brain Mapp.

[b0295] Muthukumaraswamy S.D., Singh K.D., Swettenham J.B., Jones D.K. (2010). Visual gamma oscillations and evoked responses: variability, repeatability and structural MRI correlates. Neuroimage.

[b0305] Niessing J., Ebisch B., Schmidt K.E., Niessing M., Singer W., Galuske R.A. (2005). Hemodynamic signals correlate tightly with synchronized gamma oscillations. Science.

[b0310] Nolte G. (2003). The magnetic lead field theorem in the quasi-static approximation and its use for magnetoencephalography forward calculation in realistic volume conductors. Phys Med Biol.

[b0315] Nyhus E., Curran T. (2010). Functional role of gamma and theta oscillations in episodic memory. Neurosci Biobehav Rev.

[b0320] Oostenveld R., Fries P., Maris E., Schoffelen J.M. (2011). FieldTrip: open source software for advanced analysis of MEG, EEG, and invasive electrophysiological data. Comput Intell Neurosci.

[b0325] Polman C.H., Reingold S.C., Banwell B., Clanet M., Cohen J.A., Filippi M., Fujihara K., Havrdova E., Hutchinson M., Kappos L., Lubin F.D. (2011). Diagnostic criteria for multiple sclerosis: 2010 revisions to the McDonald criteria. Ann Neurol.

[b0330] Perry G., Randle J.M., Koelewijn L., Routley B.C., Singh K.D. (2015). Linear tuning of gamma amplitude and frequency to luminance contrast: evidence from a continuous mapping paradigm. PLoS ONE.

[b0335] Petzold G.C., Murthy V.N. (2011). Role of astrocytes in neurovascular coupling. Neuron.

[b0340] R Core Team (2016). R: A language and environment for statistical computing.

[b0345] Schoonheim M.M., Meijer K.A., Geurts J.J. (2015). Network collapse and cognitive impairment in multiple sclerosis. Front Neurol.

[b0350] Singh K.D., Barnes G.R., Hillebrand A., Forde E.M., Williams A.L. (2002). Task-related changes in cortical synchronization are spatially coincident with the hemodynamic response. Neuroimage.

[b0355] Smith S.M., De Stefano N., Jenkinson M., Matthews P.M. (2001). Normalised accurate measurement of longitudinal brain change. J Comput Assist Tomogr.

[b0360] Smith S.M., Zhang Y., Jenkinson M., Chen J., Matthews P.M., Federico A., De Stefano N. (2002). Accurate, robust and automated longitudinal and cross-sectional brain change analysis. NeuroImage.

[b0365] Smith S.M. (2002). Fast robust automated brain extraction. Hum Brain Mapp.

[b0370] Smith K.J., Lassmann H. (2002). The role of nitric oxide in multiple sclerosis. Lancet Neurol.

[b0375] Sun X., Tanaka M., KoNDo S., Okamoto K., Hirai S. (1998). Clinical significance of reduced cerebral metabolism in multiple sclerosis: a combined PET and MRI study. Ann Nucl Med.

[b0380] Swank R.L., Roth J.G., Woody D.C. (1982). Cerebral blood flow and red cell delivery in normal subjects and in multiple sclerosis. Neurol Res.

[b0385] Swettenham J.B., Muthukumaraswamy S.D., Singh K.D. (2013). BOLD responses in human primary visual cortex are insensitive to substantial changes in neural activity. Front Hum Neurosci.

[b0390] Tan H.R., Gross J., Uhlhaas P.J. (2016). MEG sensor and source measures of visually induced gamma-band oscillations are highly reliable. NeuroImage.

[b0395] Tewarie P., Schoonheim M.M., Stam C.J., van der Meer M.L., van Dijk B.W., Barkhof F., Polman C.H., Hillebrand A. (2013). Cognitive and clinical dysfunction, altered MEG resting-state networks and thalamic atrophy in multiple sclerosis. PLoS ONE.

[b0400] Tewarie P., Steenwijk M.D., Tijms B.M., Daams M., Balk L.J., Stam C.J., Uitdehaag B.M., Polman C.H., Geurts J.J., Barkhof F. (2014). Disruption of structural and functional networks in long-standing multiple sclerosis. Hum Brain Mapp.

[b0405] Tewarie P., Schoonheim M.M., Schouten D.I., Polman C.H., Balk L.J., Uitdehaag B.M., Geurts J.J., Hillebrand A., Barkhof F., Stam C.J. (2015). Functional brain networks: Linking thalamic atrophy to clinical disability in multiple sclerosis, a multimodal fMRI and MEG study. Hum Brain Mapp.

[b0410] Tomassini V., Matthews P.M., Thompson A.J., Fuglø D., Geurts J.J., Johansen-Berg H., Jones D.K., Rocca M.A., Wise R.G., Barkhof F., Palace J. (2012). Neuroplasticity and functional recovery in multiple sclerosis. Nat Rev Neurol.

[b0415] Tomassini V., d'Ambrosio A., Petsas N., Wise R.G., Sbardella E., Allen M., Tona F., Fanelli F., Foster C., Carnì M., Gallo A., Pantano P., Pozzilli C. (2016). The effect of inflammation and its reduction on brain plasticity in multiple sclerosis: MRI evidence. Hum Brain Mapp.

[b0420] Toosy A.T., Mason D.F., Miller D.H. (2014). Optic neuritis. Lancet Neurol.

[b0425] Tzourio-Mazoyer N., Landeau B., Papathanassiou D., Crivello F., Etard O., Delcroix N., Mazoyer B., Joliot M. (2002). Automated anatomical labeling of activations in SPM using a macroscopic anatomical parcellation of the MNI MRI single-subject brain. Neuroimage.

[b0430] Uhlhaas P.J., Singer W. (2006). Neural synchrony in brain disorders: relevance for cognitive dysfunctions and pathophysiology. Neuron.

[b0435] Uzuner G.T., Uzuner N. (2016). Neurovascular coupling in patients with relapsing-remitting multiple sclerosis. Clin Neurol Neurosurg.

[b0440] Van der Meer M., Tewarie P., Schoonheim M., Douw L., Barkhof F., Polman C., Stam C., Hillebrand A. (2013). Cognition in MS correlates with resting-state oscillatory brain activity: An explorative MEG source-space study. NeuroImage Clin.

[b0445] Vidal-Jordana A., Sastre-Garriga J., Pérez-Miralles F., Pareto D., Rio J., Auger C., Tintoré M., Rovira A., Montalban (2016). Brain Volume Loss During the First Year of Interferon-Beta Treatment in Multiple Sclerosis: Baseline Inflammation and Regional Brain Volume Dynamics. J Neuroimaging.

[b0450] Warnert E.A., Harris A.D., Murphy K., Saxena N., Tailor N., Jenkins N.S., Hall J.E., Wise R.G. (2014). In vivo assessment of human brainstem cerebrovascular function: a multi-inversion time pulsed arterial spin labelling study. J Cereb Blood Flow Metab.

[b0455] Werring D.J., Bullmore E.T., Toosy A.T., Miller D.H., Barker G.J., MacManus D.G., Brammer M.J., Giampietro V.P., Brusa A., Brex P.A., Moseley I.F., Plant G.T., McDonald W.I., Thompson A.J. (2000). Recovery from optic neuritis is associated with a change in the distribution of cerebral response to visual stimulation: a functional magnetic resonance imaging study. J Neurol Neurosurg Psychiatry.

[b0460] Whittaker J.R., Driver I.D., Bright M.G., Murphy K. (2016). The absolute CBF response to activation is preserved during elevated perfusion: Implications for neurovascular coupling measures. Neuroimage.

[b0465] Wong E.C., Buxton R.B., Frank L.R. (1998). Quantitative imaging of perfusion using a single subtraction (QUIPSS and QUIPSS II). Magn Reson Med.

[b0470] Worsley, KJ (2001), Statistical analysis of activation images. In: Lewin JS (2003), Functional MRI: an introduction to methods. J Magn Reson Imaging 17(3), 251–270.

[b0475] Zhang Y., Brady M., Smith S. (2001). Segmentation of brain MR images through a hidden Markov random field model and the expectation-maximization algorithm. IEEE Trans Med Imag.

[b0480] Zhu Z., Zumer J.M., Lowenthal M.E., Padberg J., Recanzone G.H., Krubitzer L.A., Nagarajan S.S., Disbrow E.A. (2009). The relationship between magnetic and electrophysiological responses to complex tactile stimuli. BMC Neurosci.

[b0485] Zivadinov R., Reder A.T., Filippi M., Minagar A., Stüve O., Lassmann H., Racke M.K., Dwyer M.G., Frohman E.M., Khan O. (2008). Mechanisms of action of disease-modifying agents and brain volume changes in multiple sclerosis. Neurology.

[b0490] Zumer J.M., Brookes M.J., Stevenson C.M., Francis S.T., Morris P.G. (2010). Relating BOLD fMRI and neural oscillations through convolution and optimal linear weighting. Neuroimage.

